# Brain activity associated with Dual‐task performance of Ankle motor control during cognitive challenge

**DOI:** 10.1002/brb3.1349

**Published:** 2019-07-02

**Authors:** Sue Peters, Janice J. Eng, Teresa Liu‐Ambrose, Michael R. Borich, Elizabeth Dao, Ameen Amanian, Lara A. Boyd

**Affiliations:** ^1^ Faculty of Medicine, Department of Physical Therapy University of British Columbia Vancouver British Columbia Canada; ^2^ Djavad Mowafaghian Centre for Brain Health University of British Columbia Vancouver British Columbia Canada; ^3^ School of Medicine, Division of Physical Therapy Emory University Atlanta Georgia; ^4^ Graduate Program in Rehabilitation Sciences University of British Columbia Vancouver British Columbia Canada; ^5^ Faculty of Applied Science, Department of Electrical and Computer Engineering University of British Columbia Vancouver British Columbia Canada

**Keywords:** attention, brain, cognition, cognitive‐motor Dual tasking, Flanker, fMRI, somatosensory

## Abstract

**Introduction:**

Skilled Ankle motor control is frequently required while performing secondary cognitively demanding tasks such as socializing and avoiding obstacles while walking, termed “Dual tasking.” It is likely that Dual‐task performance increases demand on the brain, as both motor and cognitive systems require neural resources. The purpose of this study was to use functional MRI to understand which brain regions are involved in resolving Dual‐task interference created by requiring high levels of Ankle motor control during a cognitive task.

**Methods:**

Using functional MRI, brain activity was measured in sixteen young adults during performance of visually cued Ankle plantar flexion to a target (Ankle task), a cognitive task (Flanker task), and both tasks simultaneously (Dual task).

**Results:**

Dual‐task performance did not impact the Ankle task (*p* = 0.78), but did affect behavior on the Flanker task. Response times for both the congruent and incongruent conditions during the Flanker task were significantly longer (*p* < 0.001, *p* = 0.050, respectively), and accuracy for the congruent condition decreased during Dual tasking (*p* < 0.001). Activity in 3 brain regions was associated with Dual‐task Flanker performance. Percent signal change from baseline in Brodmann area (BA) 5, BA6, and the left caudate correlated with performance on the Flanker task during the Dual‐task condition (*R^2^* = 0.261, *p* = 0.04; *R^2^* = −0.258, *p* = 0.04; *R^2^* = 0.303, *p* = 0.03, respectively).

**Conclusions:**

Performance of Ankle motor control may be prioritized over a cognitive task during Dual‐task performance. Our work advances Dual‐task research by elucidating patterns of whole brain activity for Dual tasks that require Ankle motor control during a cognitive task.

## 1 INTRODUCTION

A high level of Ankle motor control is required for tasks such as driving a vehicle and locomotion over perilous terrain like snow and ice. Safe and efficient Ankle motor control in demanding environmental situations also depends on cognition. In daily life, skilled Ankle motor control is frequently required while performing secondary cognitively demanding tasks such as socializing and avoiding obstacles while walking, termed “Dual tasking” (Liu‐Ambrose, Pang, & Eng, 2007). Past functional magnetic resonance imaging (fMRI) investigations during Dual tasking focused on the behavior and patterns of brain activation associated with *hand* motor and cognitive tasks (Akkal, Bioulac, Audin, & Burbaud, 2002; Fan, Flombaum, McCandliss, Thomas, & Posner, 2003; Leone et al., 2017; Poldrack et al., 2005; Schubert & Szameitat, 2003). Yet, whole brain activity supporting *Ankle* motor control under cognitively demanding conditions has not been studied even though it may contribute to the success or failure of real‐world Dual tasks.

As a *Single task*, proprioceptive matching of Ankle plantar flexion produces activity in the primary motor (M1), primary sensory (S1), supplementary motor area (SMA), premotor, and inferior/superior parietal lobules (Iandolo et al., 2015). When performing Ankle movements without a proprioceptive requirement, similar regions are active in addition to subcortical areas including cerebellum, thalamus, caudate, and putamen (Ciccarelli et al., 2005; Cunningham, Machado, Yue, Carey, & Plow, 2013; Jaeger et al., 2014; Sahyoun, Floyer‐Lea, Johansen‐Berg, & Matthews, 2004). As a *Dual task*, Johannsen et al. (2013) examined slow, auditory‐cued Ankle movements during a working memory task (Johannsen et al., 2013). Similar to previous reports, the authors found increased brain activity in several motor regions; however, it is unclear which brain regions participated in resolving sensorimotor conflict or Dual‐task interference from the working memory task. Thus, subcortical areas and the interaction with cortical regions may be critical to successful performance of Dual tasks that require high levels of Ankle motor control.

*Interference* occurs when there is a decrement in performance of a task and when the information required to complete it conflicts with another goal. A common cognitive paradigm that elicits interference is the Eriksen Flanker task (Chen et al., 2015; Colcombe et al., 2004; Eriksen & Eriksen, 1974; von der Gablentz, Tempelmann, Munte, & Heldmann, 2015; Liu et al., 2017). Brain regions activated during the Flanker task include the precuneus, the anterior cingulate, superior/middle/inferior frontal gyrus, inferior/superior parietal lobule, and others (Berron, Fruhholz, & Herrmann, 2015; Fan et al., 2003). Functional MRI (fMRI) studies show that performing a Dual‐task leads to “over‐additive” activation of brain regions during Dual‐task performance. This “over‐additive” activation can be seen in *regions already active* during Single‐task performance. The resulting interference and decreased performance are a result of task demands that exceed the capacity of the brain regions that were initially active (Blumen, Holtzer, Brown, Gazes, & Verghese, 2014; Leone et al., 2017; Nijboer, Borst, van Rijn, & Taatgen, 2014; Schubert & Szameitat, 2003; Wu, Liu, Hallett, Zheng, & Chan, 2013). Also, *other brain regions may become active* signifying that certain regions function specifically for managing Dual‐task interference (Gruber, 2001; Johannsen et al., 2013; Leone et al., 2017).

Similarly, Dual tasks that involve visual/auditory tasks with hand responses (i.e., not Ankle responses) show an increase in activity in regions such as the prefrontal and inferior frontal sulcus (IFS) to coordinate the concurrently performed tasks (Szameitat, Schubert, Muller, & Cramon, 2002). The Dual task‐related activation in the IFS reflects an increase in the need to manage interfering information to determine the appropriate action (Schubert & Szameitat, 2003). In other words, the IFS is thought to be a region that controls Dual‐task interference (Stelzel, Schumacher, Schubert, & D'Esposito, 2006). To date, whether these regions are involved with resolving the interference of a Dual task that demands high levels of Ankle motor control is unknown. Thus, the purpose of the current study was to use fMRI to understand which regions are involved in resolving Dual‐task interference associated with Ankle motor control during the Flanker cognitive task. Second, we sought to establish a more direct link between neural correlates and motor performance; relevant brain regions active during Dual tasking were subjected to correlational analysis with behavioral data. We hypothesized that during Dual‐task conditions, behavioral performance for the cognitive task would decrease and that activity in regions of the brain supporting attention, conflict, and sensorimotor processing would correlate with behavioral parameters.

## 2 METHODS

### 2.1 Participants

Twenty‐two young, healthy volunteers gave informed written consent in accordance with the Declaration of Helsinki to participate in the study. The *University of British Columbia Ethics Review Board* approved all study procedures under ethics certificate H09‐00504. All participants were free from medical impairment or disease, had normal vision, and were right hand dominant (as determined by the Edinburgh Handedness Inventory; Oldfield, 1971). Standard magnetic resonance imaging (MRI) exclusion criteria were used.

### 2.2 Functional (f) MRI tasks

Six, 7‐min runs of functional data were collected (210 images acquired in each run). Each run contained three periods of rest (20 images each) interleaved with periods of responding (40 images each). Two runs of each Single task (i.e., Flanker alone, Ankle alone) and two runs of the Dual‐task condition (i.e., Flanker with Ankle) were performed for a total of six runs; the order of the runs was counterbalanced across participants. For both the Dual and Single tasks, the timing of the presentation of the visual stimulus was the same so that the coupling of the two tasks during the Dual Task was equivalent to the Single Tasks.

#### 2.2.1 “Ankle” task

Controlled Ankle plantar flexion movements were performed as a magnetic resonance (MR)‐compatible task. The peak pressure across the three left foot plantar flexion trials was used as a measure of maximal voluntary contraction (MVC), and participants performed plantar flexion of the left foot to a target at 30% MVC (condition termed “Ankle”).

#### 2.2.2 “Flanker” task

We employed the Eriksen Flanker task as our cognitively challenging behavior (Chen et al., 2015; Colcombe et al., 2004; Eriksen & Eriksen, 1974; von der Gablentz et al., 2015; Liu et al., 2017). This task requires identification of congruence between stimuli concurrently presented (condition termed “Flanker”). Participants were required to indicate via keypress with either the index (congruent) or middle finger (incongruent) of their left hand to indicate the congruence of the central arrow with the Flanker arrows. During the Dual task, blocks of the “Ankle” task were performed during the Eriksen Flanker task.

### 2.3 Data analysis and preprocessing

All fMRI data processing was performed using Analysis of Functional NeuroImages (AFNI) software (Cox, 1996). Data from two individuals were excluded owing to excessive head motion (>4 mm translation and/or >3° rotation) during task performance, and data from four individuals were lost owing to technical issues during data collection. Thus, sixteen individuals were included in the analysis (eight females, mean age 29.3 ± 7.7 years [±*SD*]).

### 2.4 Statistical analysis

#### 2.4.1 fMRI data

After preprocessing, a random effects general linear model (GLM) was employed. The model consisted of three predictors that corresponded to the three experimental conditions performed: (a) Flanker, (b) Ankle, and (c) Dual task (Flanker with Ankle). The results of the GLM analysis provided baseline coefficients for each functional run for each participant. The PSC was estimated on a voxel‐wise level across the whole brain, for each participant. A contrast analysis of variance (ANOVA) was carried out between the Dual‐task condition and the two Single‐task conditions (Dual task − (Single‐task Flanker + Single‐task Ankle)) with significance set at *p* < 0.005 together with minimum cluster size of >200 mm^3^. For the Single tasks, a similar contrast analysis identified clusters uniquely active for each Single task not present in the Dual task or the remaining Single task (e.g., Single‐task Flanker − (Dual task + Single‐task Ankle)). Significance of voxel activation was set at *p* < 0.005. The anatomical location associated with the center of mass of each cluster was identified with the use of an automated online atlas.

#### 2.4.2 Brain activation and Behavioral data analysis

Dependent measures included (a) for the “Flanker,” response time of correct trials (RT), percent correct (% accuracy), and interference score, and (b) for the “Ankle,” accuracy calculated as the force produced compared to the target force level, and (c) percent signal change (PSC) in blood oxygen level dependent (BOLD) during Dual‐task performance as compared each Single‐task condition. Mean and standard deviation (*SD*) of the RT across trials were obtained from performance of the Flanker and Ankle tasks alone. For the Ankle task, the mean and *SD* of accuracy (V) were calculated for the task alone, and during the Flanker task. Dual‐task data for both the Flanker and Ankle tasks were averaged by condition for each participant. The interference score was calculated using only correct responses on the Flanker task where: (Incongruent RT − Congruent RT)/Congruent RT (Nagamatsu, Boyd, Hsu, Handy, & Liu‐Ambrose, 2013). Paired *t* tests were conducted comparing Single tasks to Dual‐task conditions. For the Dual task only, Pearson correlation coefficients were calculated between performance (six variables: Ankle ACC, Flanker Congruent/Incongruent RT/ACC, Flanker interference score) and PSC during the Dual task identified by the ANOVA (regions in Table 2; *p* < 0.05).

## 3 RESULTS

Behavioral performance of accuracy, RT, and interference is reported in Table 1. For the Flanker task, RTs for both the congruent and incongruent conditions were significantly longer during Dual‐task performance (*t*_15_ = −8.3, *p* < 0.001; *t*_15_ = 2.1, *p* = 0.050, respectively). Accuracy, however, differed only for the congruent condition where performance worsened while Dual tasking (*t*_15_ = −4.9, *p* < 0.001). The interference score increased during the Dual task (*t*_15_ = 4.7, *p* < 0.001).

**Table 1:** Single and Dual task behavioral data

	Ankle task	Flanker task	Dual task	*p* Value
Ankle ACC (V)	0.19 ± 0.45	–	0.18 ± 0.52	0.78
Congruent RT (s)	–	0.36 ± 0.04	0.39 ± 0.05	<0.001*
Congruent ACC (%)	–	83.32 ± 8.79	73.51 ± 4.01	<0.001*
Incongruent RT (s)	–	0.39 ± 0.05	0.42 ± 0.05	0.050*
Incongruent ACC (%)	–	68.57 ± 12.34	65.22 ± 9.78	0.14
Interference score	–	−0.04 ± 0.16	0.08 ± 0.04	<0.001*

Note

Values are mean ± standard deviation.

Abbreviations: ACC, accuracy; RT, response time.

* Statistically significant difference (*p* ≤ 0.05).

The results of the contrast ANOVA indicated that 18 regions were uniquely active in the Dual‐task condition (vs. the two Single‐ task conditions: Table 2). Active brain regions during the Dual‐task condition included the bilateral precuneus, paracentral lobule, posterior cingulate, and the cerebellum (Table 2). For the Flanker and Ankle Single tasks, a similar contrast analysis identified 12 and 14 regions, respectively, uniquely active for each Single task not present in the Dual task or the remaining Single task (Table 2). During the “Flanker,” increased activity in the precuneus, anterior cingulate and bilateral motor cingulate, and the parahippocampal gyri were found (Table 2). During the “Ankle” condition, regions included the anterior, posterior, and motor cingulate, precuneus, and cuneus (Table 2).

**Table 2:** Clusters showing unique activation per condition

Cluster volume (mm^3^)	MNI coordinates			Brain region	BA
	*X*	*Y*	*Z*		
Flanker					
16,456	2.4	−47	48.4	Precuneus (R)	7
12,993	1.5	30.5	10.2	Anterior Cingulate (R)	24
4,343	−8.6	−43.7	3.4	Parahippocampal Gyrus (L)	30
1,671	−4.2	−53	−26.1	Anterior Lobe Cerebellum (L)	a
1,147	16.5	−50.1	22.1	Cingulate Gyrus (R)	31
670	9.6	64.1	27.2	Superior Frontal Gyrus (R)	10
624	−8.3	23.4	36.4	Cingulate Gyrus (L)	32
561	2.9	50	34.7	Medial Frontal Gyrus (R)	9
503	11.3	−40.9	69.8	Paracentral Lobule (R)	4
379	24.3	−70.5	33.9	Precuneus (R)	7
211	−24.8	−86.4	3.8	Middle Occipital Gyrus (L)	18
207	45.4	1.6	−11.3	Superior Temporal Gyrus (R)	38
Ankle					
7,993	6.1	−47.9	21.1	Posterior Cingulate (R)	30
5,873	15.9	−41.4	51.9	Precuneus (R)	7
863	−2	3	25.1	Cingulate (L)	24
674	1.4	−76.3	18.6	Cuneus (R)	18
611	1.5	3	−4.8	Anterior Cingulate (R)	25
542	−11.1	26	15.8	Anterior Cingulate (L)	24
523	−59.7	−35.7	6.2	Superior Temporal Gyrus (L)	22
401	12.2	24.6	14.6	Caudate (R)	a
316	0.9	−57.8	−27	Anterior lobe Cerebellum (R)	a
282	−19.1	−38.8	5.8	Parahippocampal Gyrus (L)	30
264	−4.5	−27.5	32.1	Cingulate (L)	23
259	−49.6	−52.2	27.9	Supramarginal Gyrus (L)	40
240	−12.6	−65.3	49.1	Precuneus (L)	7
210	13.1	50.8	27.8	Superior Frontal Gyrus (R)	9
Dual task					
4,940	−8.5	−42.4	57.1	Paracentral Lobule (L)	5
2,396	0.9	−71.1	23.5	Precuneus (L)	31
1,293	3.5	−42.1	16.6	Posterior Cingulate (R)	29
1,173	18.9	−66.7	46.1	Precuneus (R)	7
1,097	24.4	−41.9	−27	Anterior Lobe Cerebellum (R)	a
804	13.1	−39.1	53.5	Precuneus (R)	7
752	−4.6	−17.7	60.3	Medial Frontal Gyrus (L)	6
700	−29.9	1	−19.2	Parahippocampal Gyrus (L)	34
524	22.7	18.5	38.2	Middle Frontal Gyrus (R)	8
474	−32.1	20	42	Middle Frontal Gyrus (L)	8
452	−38	6.6	0.9	Insula (L)	13
435	45.4	−1.2	47.4	Precentral Gyrus (R)	6
418	−11.2	−69.1	36.5	Precuneus (L)	7
416	−18.7	−36.2	6.2	Thalamus (L)	30
335	−23.3	−28.9	26.3	Caudate body (L)	a
277	10.7	−56.1	6.8	Posterior Cingulate (R)	30
244	57.5	−57.4	22	Superior Temporal Gyrus (R)	39
234	−22.5	−69.8	54.7	Precuneus (L)	7

a No Brodmann area associated with cluster.

### 3.1 Dual task behavioral performance and the relationship to brain activity

**Figure 1:**
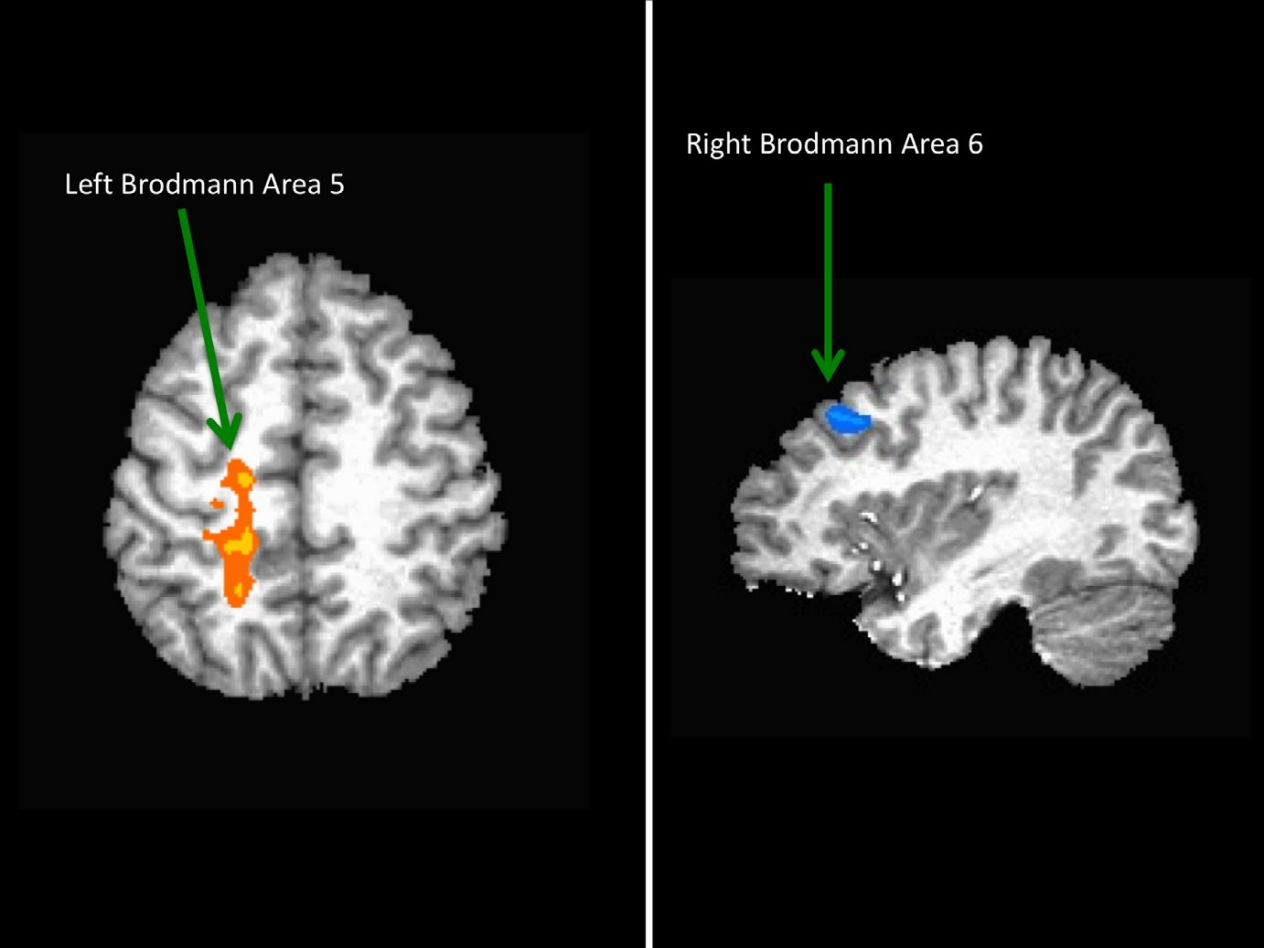
Brain activity during Dual tasking. Regions of activation included Brodmann areas 5 and 6

**Figure 2:**
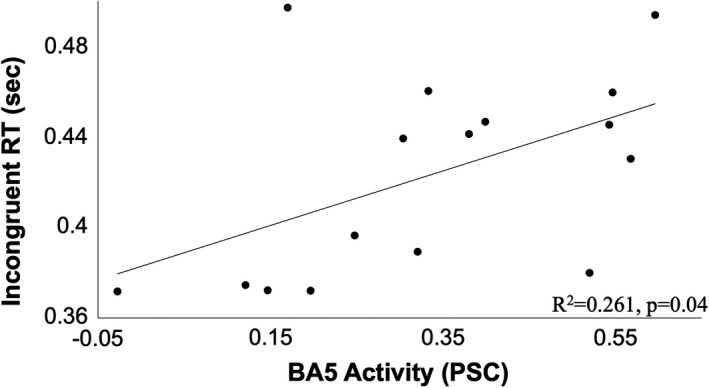
Relationship of percent signal change in Brodmann area 5 with response time on incongruent trials during the Dual task. Greater activity in Brodmann area 5 was linked with longer response times on the incongruent trials (*R* ^2^ = 0.261, *p* = 0.04)

**Figure 3:**
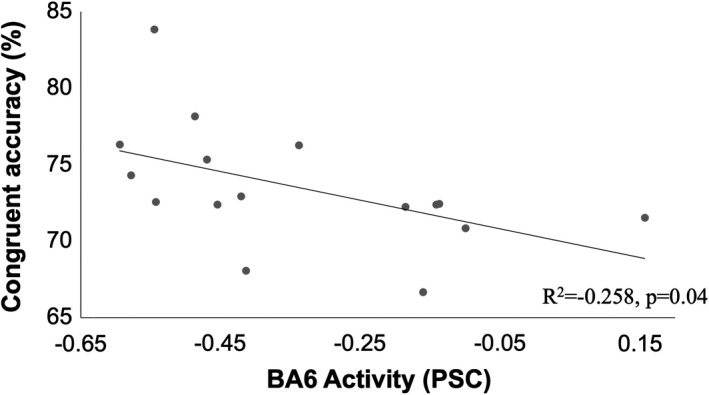
Relationship of percent signal change in Brodmann area 6 with congruent trial accuracy during the Dual task. Greater activity in Brodmann area 6 linked with reduced accuracy on congruent trials (*R* ^2^ = −0.258, *p* = 0.04)

**Figure 4:**
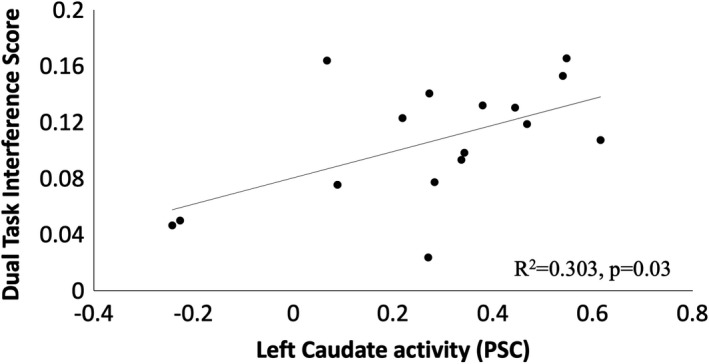
Relationship of percent signal change in the left caudate with interference score during the Dual task. Greater activity in the left caudate linked with higher interference scores (*R^2^* = 0.303, *p* = 0.027)

Three brain regions demonstrated an association with Dual‐task performance. Left Brodmann area (BA) 5, right BA6, and the left caudate were active during the Dual‐task condition and correlated with performance (group mean PSC = 0.34 ± 0.19%; −0.34 ± 0.22; 0.28 ± 0.25%, respectively; Figure 1). Activity in the left paracentral lobule (BA5) was positively correlated with RT for incongruent trials during Dual tasking (*r* = 0.511, *p* = 0.04, Figure 2). Greater activity in BA5 was related to slower performance in incongruent trials. For congruent trials, however, greater accuracy was associated with negative PSC values in the right precentral gyrus (BA6; *r* = −0.508, *p* = 0.04, Figure 3). Increased activity in the left caudate corresponded to a greater interference score during the Dual task (*r* = 0.550, *p* = 0.03; Figure 4). A significant relationship was not observed between brain activity and behavioral measures of Ankle performance during the Dual‐ task condition.

## 4 DISCUSSION

### 4.1 Summary of main findings

Under Dual‐task conditions, cognitive performance decreased, whereas Ankle performance was unchanged indicating prioritization of Ankle motor control over the cognitive task. This is consistent with the decline in cognitive performance during obstacle crossing in young adults (Siu, Catena, Chou, van Donkelaar, & Woollacott, 2008) and suggests that decreases in cognitive performance may be associated with the complexity of the cognitive task being performed (Kelly, Eusterbrock, & Shumway‐Cook, 2013; Patel, Lamar, & Bhatt, 2014). What is interesting is that in our experimental paradigm, there was no potential for a fall; yet, study participants still prioritized Ankle motor control over the cognitive task. Also, relatively simple motor tasks must be designed due to the constraints of fMRI, so that it is possible that see declines in motor performance are not observed. Regardless, our work advances Dual‐task research by elucidating patterns of whole brain activity for Dual tasks that require Ankle motor control during a cognitive task. Increased activation was present in brain areas associated with motor performance and learning (caudate), motor planning (BA6), and somatosensory and conflict information processing (BA5; Carbon et al., 2004).

### 4.2 Resolving somatosensory conflict during Dual tasking

Relationships between brain activity in BA6 and cognitive performance suggest that during a Dual task with less cognitively demanding conditions (i.e., congruent trials), young adults who demonstrate higher task accuracy may need less cortical activity for motor planning. This was noted by the lower activity in BA6 associated with congruent responses during Dual tasking. It is possible that the reduced cognitive load translates into less brain activity in motor planning regions since the motor response does not require the resolution of sensorimotor conflict prior to motor plan formation.

Increased time to accurately respond to incongruent trials during Dual tasking suggest that a delayed motor response may be linked to increased somatosensory processing in BA5 and associated with increased cognitive task complexity (Figure 2). In other words, greater processing effort for resolving conflicting visual stimuli (incongruency in the Flanker task) requires more time to produce coordinated motor output. Additionally, the lower response accuracy for trials with sensory conflict suggests that despite participants taking longer to resolve conflict, the demand for a quick response is prioritized over accuracy (Table 1). The additive effects of increased time to respond together with reduced accuracy have functional implications for those with difficulty doing two things at once, such as walking while talking.

### 4.3 Learning to resolve somatosensory conflict

Our work discovered that activity in one subcortical region, the caudate body, related to Dual‐task interference (Figure 4). Previous research noted a relationship between activity in the caudate and sequence learning using the upper extremity (Carbon et al., 2004). Additionally, reduced activity in the left caudate nucleus has been associated with increasing automaticity of motor performance when learning right hand and finger sequences (Wu, Kansaku, & Hallett, 2004). When learning to resolve stimulus–response conflict, such as is present in the Flanker task, the caudate is involved with altering “decision criteria” on a trial‐by‐trial basis (Berron et al., 2015). Improvements in conflict processing could facilitate enhanced motor performance and ultimately result in motor learning if a similar type of stimulus–response task is practiced. The link between greater activity in the caudate and higher interference scores during the Dual task (Figure 4) suggests that learning a Dual Ankle motor control/cognitive task can influence motor performance. Further work examining which specific learning strategies may reduce the cognitive load of Ankle motor control during cognitive challenge is warranted in an aging or clinical population, as it may have the potential to reduce Dual task‐related falls.

### 4.4 Limitations

Ankle motor control paradigms that are fMRI compatible must be performed in supine, where falls are not possible and as such, are limited in applicability to upright walking. However, Ankle motor control tasks during fMRI do reveal that the contralateral primary motor, supplementary motor/premotor, primary sensory, cingulate, and cerebellum are engaged and described as a proxy for walking motor control (Dobkin, Firestine, West, Saremi, & Woods, 2004).

Some research indicates that Dual tasking results in decreased motor and cognitive performance (Melzer, Benjuya, & Kaplanski, 2001; Mitra, Knight, & Munn, 2013; Weeks, Forget, Mouchnino, Gravel, & Bourbonnais, 2003). As in other studies of Dual tasking using fMRI, there are constraints in the selection of motor tasks, limiting researchers to more simple motor tasks (Francis et al., 2009; MacIntosh et al., 2004; Schubert & Szameitat, 2003; Stelzel et al., 2006; Szameitat et al., 2002). The Ankle motor control task utilized in this study may not be challenging enough to produce a decrease in motor performance during the Dual task, which future studies can address by comparing our Ankle task to one with additional speed or accuracy requirements.

While our sample size is consistent with other fMRI work involving Ankle motor control (Dobkin et al., 2004; MacIntosh et al., 2004; Newton et al., 2008), we did not correct for multiple comparisons due to the exploratory nature of this study. Further research is warranted with older adults to determine the influence of aging on Dual tasking as this cohort is more likely to have reduced motor performance potentially resulting in increased fall risk. Falls represent the largest source of injury in older adults (Centers for Disease Control & Prevention, 2006). The most commonly reported environmental factors contributing to falls in older adults are uneven terrain, altered lighting, or obstacles to ambulation (Talbot, Musiol, Witham, & Metter, 2005). It stands to reason that Ankle motor control is an important ability to maintain with aging for safe community living. Understanding the neural correlates of performing skilled motor control of the Ankle together with cognitively demanding tasks is critical to the development of novel interventions designed to prevent falls in older individuals.

### 4.5 Conclusions

This study is the first to characterize whole brain activity patterns during Dual‐task performance of Ankle motor control with a cognitive task. We noted that Dual‐task performance did not impact the Ankle task but did affect performance of the Flanker task, though our Ankle task may have been too simple to affect Dual‐task performance. This implies that under Dual‐task conditions, when young adults need to perform both Ankle motor control and cognitively challenging tasks, motor task performance is prioritized. However, testing a more difficult task with a larger sample size may increase granularity of these findings. An essential next step in this line of research is to determine whether Dual‐task *training* might enhance the simultaneous performance of Ankle motor control and cognitive tasks. Findings from these investigations may have important rehabilitation implications that can be examined in future studies in clinical populations.

## DATA AVAILABILITY STATEMENT

The data that support the findings of this study are available from the corresponding author upon reasonable request.
