# Supplementary material for: Brain activity associated with Dual‐task performance of Ankle motor control during cognitive challenge
Source: Brain Behav. 2019 Jul 2;9(8):e01349. doi: 10.1002/brb3.1349 (PMC6710191; doi:10.1002/brb3.1349)
Supplement: Supplementary file 1 [file BRB3-9-e01349-s001.docx]

Supplementary Materials – Background, Replicability, and Discussion

Introduction

There are few theories regarding the neurophysiology of brain activation associated with fMRI. The reader is directed to the following references for further reading:

Raichle, M. E., & Gusnard, D. A. (2002). Appraising the brain's energy budget. *Proc Natl Acad Sci U S A, 99*(16), 10237-10239.

Zauner, A., Daugherty, W. P., Bullock, M. R., & Warner, D. S. (2002). Brain oxygenation and energy metabolism: part I-biological function and pathophysiology. *Neurosurgery, 51*(2), 289-301; discussion 302.

Methods

***fMRI tasks:*** Controlled ankle plantarflexion movements were performed as a magnetic resonance (MR)-compatible task. A custom MR-compatible air-based bulb apparatus was used to measure pressure of the plantarflexion movement produced with a pedal and was connected to a pressure transducer via a PVC tube. During scanning, the pressure transducer was situated in the MRI control room. The signal from the pressure transducer was connected to a computer through a serial interface sampled by a custom LabVIEW (version 9.0.1, National Instruments) program with visual feedback given to the participant; a visual target was presented at 30% of MVC along with a visual indicator of current plantarflexion performance. The computer display controlled the timing of plantarflexion, via a back projected image on a screen visible to the participants in the MRI scanner. Stimulus presentation was time-locked with MR image acquisition.

All plantarflexion movements during the block were normalized to MVC. The angle and position of the pedal was adjusted for each participant so that the ball of the foot pushed on the pedal. Considering that a larger number and greater magnitude of activation of regions are present during bilateral ankle movement, a unilateral ankle motor control task was needed to match the unilateral hand response for the Flanker task (Noble, Eng, & Boyd, 2014). Participants were instructed to move as fast and accurately as possible for the ankle task. Participants increased pressure on the pedal until the performance indicator matched the target. Thus, the task featured a high level of spatial and temporal precision, and a low level of automaticity. Immediately prior to data collection, each individual practiced with the device and task to ensure a high level of familiarity.

For the Flanker task, the stimuli were a set of arrows (“>”) with the central arrow flanked by two additional arrows on both sides (5 total arrows) that were either in agreement (i.e., congruent) or in conflict (i.e., incongruent) with the central arrow. Participants were required to indicate via key-press with either the index (congruent) or middle finger (incongruent) of their left hand to indicate the congruence of the central arrow with the flaker arrows, and were instructed to respond as fast and accurately as possible. For the dual task, participants were instructed to respond as fast and accurately as possible for both tasks, implying equal task priority. The visual stimuli in the all tasks were time-locked to MR image acquisition.

***Data analysis and preprocessing***: Functional imaging data were collected as echo-planar images, by using a single-shot, blipped gradient-echo echo-planar pulse sequence using a BOLD contrast (TR=2.0 s, TE=30 ms, flip angle = 90°, 36 contiguous slices at 3 mm skip 1 mm, FOV=256 mm, 64 x 64). Prior to functional imaging, high-resolution T1-weighted axial images (TR=24 ms, TE=5 ms, flip angle=40°, voxel size = 1x1x1 mm, thickness=1.2 mm, FOV=256 mm) were collected for anatomic localization and co-registration of the functional images. Scanning was performed at the UBC MRI Research Centre with a whole body 3.0T Philips Achieva MRI scanner (Philips Healthcare, Andover, MD) to collect whole brain fMRI (36 axial, 3 mm slices with 1 mm gap).

All fMRI data processing was performed using Analysis of Functional NeuroImages (AFNI) software (Cox, 1996). Sixteen individuals were included in the analysis (8 females, mean age 29.3 ± 7.7 years (±SD)). First, functional data were 3D motion and slice time corrected. Time courses were then high pass filtered to remove linear trends. Following pre-processing, the functional data were transformed into standard space (Talairach & Tournoux, 1988) through co-registration with spatially transformed 3D anatomical data sets for each individual subject. The resulting volume time courses were then filtered using a 4-mm Gaussian kernel at full width half maximum.

After preprocessing, to evaluate the differences in the magnitude of the hemodynamic response across conditions a random effects general linear model (GLM) was employed. The model consisted of three predictors that corresponded to the three experimental conditions performed: (1) Flanker, (2) Ankle, and (3) Dual task (Flanker with Ankle). Six additional predictors of no interest were included to account for translational and rotational motion in the x, y, and z planes. The results of the GLM analysis provided baseline coefficients for each functional run, which defined the variation in baseline activity during the resting periods, for each participant. The PSC was estimated on a voxel-wise level across the whole brain, for each participant, by dividing the regression coefficient for the three conditions by the average of the baseline coefficients and multiplying by 100. The PSC value was calculated on a voxel-wise basis, for each participant. The analysis identified significantly active clusters during the Dual task condition, but not during either single task condition. Probability thresholding was used with minimum-cluster-size thresholding (Forman et al., 1995) to determine regions of activation (minimum cluster size >200 mm^3^).

***Flanker task interference calculation:*** The interference score was calculated during the Flanker alone and during the Dual task condition, using only correct responses on the Flanker task where: (Incongruent RT – Congruent RT)/Congruent RT (Nagamatsu, Boyd, Hsu, Handy, & Liu-Ambrose, 2013).

Discussion

***Resolving somatosensory conflict during dual tasking***: Though some studies show the inferior frontal sulcus helps resolve conflict resolution associated with interfering sensorimotor information (Schubert & Szameitat, 2003; Stelzel, Schumacher, Schubert, & D'Esposito, 2006), BA6 also plays a role in processing sensorimotor conflict and serves to resolve interference (Nachev, Kennard, & Husain, 2008). While it is possible that BA6 functions to resolve the interference present in the dual task paradigm used in our experiment, we only found a relationship between interference scores and activity in the caudate (Figure 4). In the context of the “over-additive” theory (Leone et al. 2017), if BA6 functions to plan and resolve the interfering sensorimotor information from the dual Ankle and Flanker tasks and this process extends beyond the capacity of BA6, then the caudate may increase activity to assist in managing dual task interference (Figure 4).

***The role of the cerebellum for skilled dual tasking:*** Cerebellar activity during our dual task may also indicate increased effort to control the timing, force, rate, and amplitude generation in the ankle, and the integration of both motor and cognitive demands of the dual task (Wu, Liu, Hallett, Zheng, & Chan, 2013). Additionally, our finding of a similar interplay of brain regions during the dual task condition (Table 2) suggests that ankle motor control while performing a cognitively demanding task requires closely aligned stimulus-response adaptation and performance monitoring (von der Gablentz, Tempelmann, Munte, & Heldmann, 2015). The interplay between brain regions involved with determining time to respond, as well as magnitude and accuracy of response for the dual task, likely involve complex interactions of regions and networks. Both motor and cognitive tasks involve cerebro-cerebellar circuits (Stoodley, Valera, & Schmahmann, 2012), as such, dual tasks involving both cognitive and motor demands may engage the cerebellum in a similar manner.

References

Cox, R. W. (1996). AFNI: Software for analysis and visualization of functional magnetic resonance neuroimages. *Computers and Biomedical research, 29*, 162-173.

Forman, S. D., Cohen, J. D., Fitzgerald, M., Eddy, W. F., Mintun, M. A., & Noll, D. C. (1995). Improved assessment of significant activation in functional magnetic resonance imaging (fMRI): use of a cluster-size threshold. *Magn Reson.Med., 33*(5), 636-647.

Nachev, P., Kennard, C., & Husain, M. (2008). Functional role of the supplementary and pre-supplementary motor areas. *Nat Rev Neurosci, 9*(11), 856-869. doi:10.1038/nrn2478

Nagamatsu, L. S., Boyd, L. A., Hsu, C. L., Handy, T. C., & Liu-Ambrose, T. (2013). Overall reductions in functional brain activation are associated with falls in older adults: an fMRI study. *Front Aging Neurosci, 5*, 91. doi:10.3389/fnagi.2013.00091

Noble, J. W., Eng, J. J., & Boyd, L. A. (2014). Bilateral motor tasks involve more brain regions and higher neural activation than unilateral tasks: an fMRI study. *Exp Brain Res, 232*(9), 2785-2795. doi:10.1007/s00221-014-3963-4

Schubert, T., & Szameitat, A. J. (2003). Functional neuroanatomy of interference in overlapping dual tasks: an fMRI study. *Brain Res Cogn Brain Res, 17*(3), 733-746.

Stelzel, C., Schumacher, E. H., Schubert, T., & D'Esposito, M. (2006). The neural effect of stimulus-response modality compatibility on dual-task performance: an fMRI study. *Psychol Res, 70*(6), 514-525. doi:10.1007/s00426-005-0013-7

Stoodley, C. J., Valera, E. M., & Schmahmann, J. D. (2012). Functional topography of the cerebellum for motor and cognitive tasks: an fMRI study. *Neuroimage, 59*(2), 1560-1570. doi:10.1016/j.neuroimage.2011.08.065

Talairach, J., & Tournoux, P. (1988). *Co-planar stereotaxic atlas of the human brain*. New York: Thieme.

von der Gablentz, J., Tempelmann, C., Munte, T. F., & Heldmann, M. (2015). Performance monitoring and behavioral adaptation during task switching: an fMRI study. *Neuroscience, 285*, 227-235. doi:10.1016/j.neuroscience.2014.11.024

Wu, T., Liu, J., Hallett, M., Zheng, Z., & Chan, P. (2013). Cerebellum and integration of neural networks in dual-task processing. *Neuroimage, 65*, 466-475. doi:10.1016/j.neuroimage.2012.10.004
